# Extractive electrospray ionization mass spectrometry for analytical evaluation and synthetic preparation of pharmaceutical chemicals

**DOI:** 10.3389/fphar.2023.1110900

**Published:** 2023-01-13

**Authors:** Manman Qin, Yuqing Qian, Lu Huang, Chao Zhong, Mingdong Li, Jun Yu, Huanwen Chen

**Affiliations:** ^1^ Mass Spectrometry Laboratory for BioSample Analysis, Jiangxi University of Chinese Medicine, Nanchang, Jiangxi, China; ^2^ Key Laboratory for Pharmacology and Translational Research of Traditional Chinese Medicine of Nanchang, Centre for Translational Medicine, Jiangxi University of Chinese Medicine, Nanchang, Jiangxi, China; ^3^ Jiangxi Key Laboratory of Traditional Chinese Medicine for Prevention and Treatment of Vascular Remodeling Diseases, Nanchang, Jiangxi, China; ^4^ School of Pharmacy, Jiangxi University of Chinese Medicine, Nanchang, Jiangxi, China; ^5^ Department of Cardiovascular Sciences and Centre for Metabolic Disease Research, Lewis Katz School of Medicine, Temple University, Philadelphia, PA, United States

**Keywords:** extractive electrospray ionization mass spectrometry, neutral-desorption extractive electrospray ionization mass spectrometry, internal extractive electrospray ionization mass spectrometry, pharmaceutical chemicals, analytical evaluation, synthetic preparation

## Abstract

Extraction electrospray ionization mass spectrometry (EESI-MS), due to the unique configuration of its ionization module, enables the effective ionization of trace molecules of interest in samples containing complex matrices with high sensitivity, high selectivity and high responding speed without requiring sample pretreatment, and allows high-energy molecular species to undergo specially designed reactions for advanced functionalization. The typical effects of operating conditions on the analytical performance of extraction electrospray ionization mass spectrometry for various pharmaceutical compounds, pharmaceutical preparations and herbal materials were systematically reviewed. The application prospect of extraction electrospray ionization in molecular functionalization for advanced drug discovery is also briefly introduced.

## 1 Introduction

Mass spectrometry has been applied to multi-disciplines and sub-fields related to medicine (such as material base (Ye et al., 2019), pharmacokinetics (Steuer et al., 2017), quality control (Liu and Schulz, 2021), synthesis and preparation (Mercieca et al., 2020), etc.), because mass spectrometry offers the premier performance including high sensitivity, high specificity and rapid response to identify the ingredients and compounds of interest in the real-word pharmaceutical sample mixtures with qualitative and quantitative information.

At present, pharmaceutical related laboratories often use gas chromatography-mass spectrometry (GC-MS) (Beale et al., 2018; Wang et al., 2018) and liquid chromatography-mass spectrometry (LC-MS) (Oh et al., 2021; Zhao et al., 2021), which require dedicated sample pretreatment prior to sample analysis by mass spectrometry. Usually, the sample processing protocols are complicated and takes hours to for a single run. It has been known that besides of the possible sample loss, potential contamination and material degradation, the sample pretreatment process is a key factor restricting the analysis efficiency of mass spectrometry (Chen et al., 2010). Therefore, advanced technologies for mass spectrometry analysis of real-world samples without pretreatment is highly desirable in pharmaceutical industry and research laboratories.

Extractive Electrospray Ionization Mass Spectrometry (EESI-MS) enables direct analysis of a wide variety of complex matrices without any sample pretreatment. Due to its extremely high tolerance of dirty matrix, EESI-MS has been widely used in the safety evaluation of medicine (Chen et al., 2010; Zeng et al., 2016; Zheng et al., 2022), food analysis (Deng et al., 2017; Xu et al., 2017; Lu et al., 2021b), life science (Hu et al., 2011; Ning et al., 2013; Ke et al., 2019), environmental science (Liu et al., 2011; Liu et al., 2012; Liu et al., 2020), chemical industry (Zhu et al., 2008; Li X. et al., 2013; Zhang et al., 2018) and other fields. In addition, based on EESI-MS, Neutral-Desorption Extractive Electrospray Ionization Mass Spectrometry (ND-EESI-MS) (Wu et al., 2019; Zheng et al., 2021) and Internal Extractive Electrospray Ionization Mass Spectrometry (iEESI-MS) (Lu et al., 2021a; Zheng et al., 2022) were further developed. Herein, we provide an overview of the typical effects of operation conditions on the analytical performance of EESI-MS for various pharmaceutical compounds, pharmaceutical preparations, and herbal medicine materials. The perspective of EESI-MS application in functionalization of molecules for advanced pharmaceutical chemicals is also briefly described.

## 2 The structure and ionization principle of EESI-MS

**FIGURE 1:**
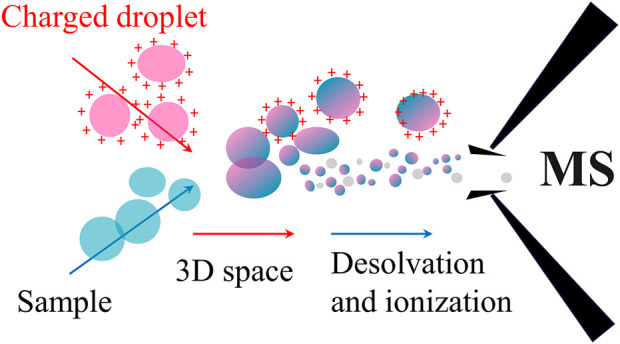
The schematic of ionization principle of EESI-MS.

**FIGURE 2:**
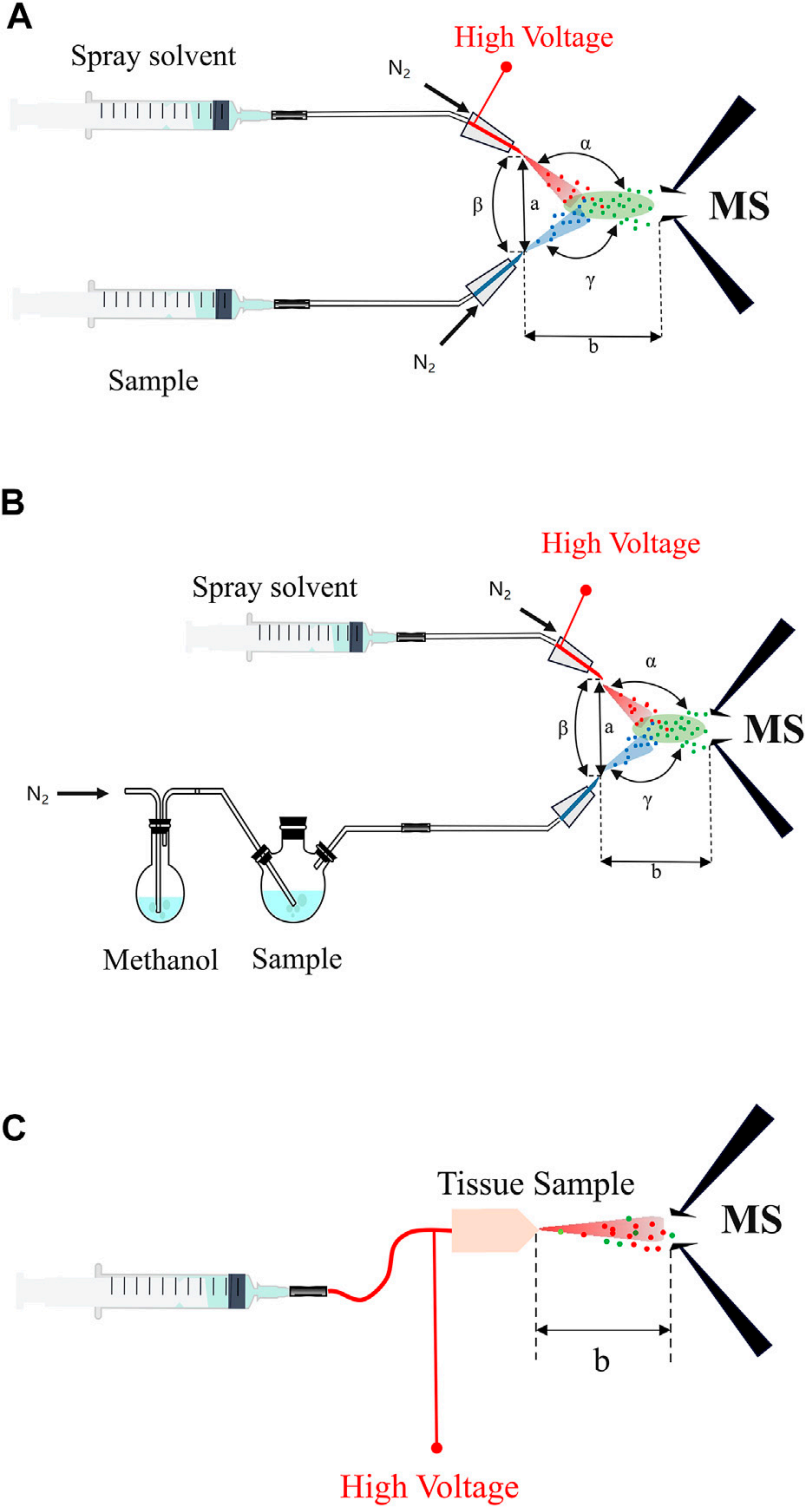
The schematic of EESI-MS and its derivatives. **(A)** The structure of EESI-MS. **(B)** The structure of ND-EESI-MS. **(C)** The structure of iEESI-MS. α: The angle between the extractant and the mass spectrum inlet; β: The angle between the two channels; γ: The angle between the sample atomizer tube and the mass spectrum inlet; a: The distance between the two channels; b: The distance between the sample atomizer tube and the mass spectrum inlet.

Traditionally, mass spectrometry ionization technologies usually require multi-step sample pretreatment before the processed sample can be introduced for ionization and mass analysis and detection in the vacuum (Chen et al., 2010; Meng, 2022). Nevertheless, EESI-MS firstly charges the selected reagent (methanol or water, etc.) using ESI for good production of the ionic reagent plume, which then implact the sample plume in the open-air with a large volume about 20 cm^3^ to transfer the energetic charge to the analyte molecules originated from the sample mixture. Subsequently, the analytes become ions through extraction and collision during the interations of the two plume sprays, and then the analytes are introduced into the mass spectrometer for subsequent analysis (Figure 1) (Chen et al., 2006). Typically, the EESI device is mainly composed of two parts: An electrospray channel and a neutral sample spray channel crossed at a certain angle (Figure 2A). By adjusting the angle (α) between the extractant and the mass spectrum inlet, the angle (β) between the two channels, the angle (γ) between the sample atomizer tube and the mass spectrum inlet, the distance (a) between the two channels and the distance (b) between the sample atomizer tube and the mass spectrum inlet, the detection limits for some substances (such as nicotine) can be even much better than ESI under the experimental conditions, probably because the overall efficiency for the ion collection, desolvation and transmission is improved than the case employing ESI. ND-EESI-MS is developed based on EESI, by adding appropriate neutral gas plume such as air, nitrogen to liberate analytes from viscous samples (i.g., human skin, honey, etc.). Usually, ND-EESI-MS is used for remote analysis by separating the sampling and ionization processes in time and space (Figure 2B) (Chen et al., 2010), allowing no/minimal invasion of the sample during the analysis. iEESI-MS directly introduces a charged solution (such as methanol, water, acetonitrile, etc.) into a large bulk sample, in which the solvent diffuses, and then the analyte solution moves along the electric field gradient inside the sample to the mass spectrometer. As a result, it forms a stable electrospray plume in front of the mass instrument entrance to produce analyte ions (Figure 2C) (Zhang et al., 2013; Xu and Chen, 2018; Lu et al., 2020).

In drug discovery, ESI-MS has been widely used in many fields such as organic synthesis, pharmaceutical analysis, and reaction monitoring. However, the ESI-MS requires specially designed multi-step pre-processing (e.g., extraction, separation, dilution, etc.), which may delay the process of analysis for several minutes or hours (Dell‘Orco et al., 1999; Brum et al., 2001; Clinton et al., 2005). Direct real-time analysis (DART) uses tubes to directly absorb solution from the reaction system, and then puts in front of a heated DART ionization source. After the solvent evaporates, the analyte was ionized and then directed to a mass spectrometer for analysis. However, high temperature heating may lead to degradation of the compound (Petucci et al., 2007). EESI-MS offers more advantages than the above mentioned technologies because it uses two independent sprays: One sprays the sample solution, and the other generates charged solvent droplets (Chen et al., 2005a; Chen et al., 2007a). This method allows directly trace detection of the analytes in the form of gas, liquid and aerosol quickly and without any sample pretreatment, and is not interfered by complex matrices (Chen et al., 2006; Chen et al., 2007a; Chen et al., 2007b; Chen et al., 2007c; Gu et al., 2007; Zhou et al., 2007; Chingin et al., 2008). Therefore, EESI-MS is suitable for real-time monitoring of chemical reaction process, and can directly detect various intermediates and by-products in the whole reaction process (Qian et al., 2005; Mothana and Boyd, 2007).

Typical features of EESI-MS are summarized as follows (Zhou et al., 2007; Zhang et al., 2013; Xu et al., 2017; Zhang et al., 2021):

(1) Rapidness: The molecular information of the sample can be obtained within few seconds (such as acetone in exhaled gas) without sample pretreatment. The capability of EESI-MS for *in vivo* analysis is a unique feature making EESI-MS outstanding among all the mass spectrometry-based technologies, and thus enables the technique attractive for life investigation at the molecular levers in real time;
(2) Sensitivity: All parameters are adjustable so that 1) the detection limit of some substances (atrazine, etc.) can reach fg level; 2) the signal is stable with RSD below 5% in the order of pg levels;
(3) Selectivity: Samples with polar, weak polar, and nonpolar properties can all be detected by altering the extraction solution;
(4) Accuracy: 1) EESI-MS allows for the analysis of the sample to obtain abundant molecular information, which may be lost during sample pretreatment by other analytical methods; 2) EESI provides maximum assurance that samples are not affected by reagents and operating conditions during mass spectrometry;
(5) Application: EESI can directly ionize liquid, colloid, gaseous, solid, powder and other samples without sample pretreatment;
(6) Automation: Precision-machined EESI is an automated sampling device that can provide high accuracy and precision.

## 3 Application of EESI-MS in analysis of pharmaceutical chemicals

EESI-MS can be used for the identification and separation of various bioactive substances, such as alkaloids, amino acids, nucleotides, polypeptides, glycosides, steroidal compounds, volatile oils, and fatty acids. It has been well established that the ionization intensity of the compound is positively correlated with the detection capability and the sensitivity of the mass spectrometer. According to the molecular structure of drugs, Chen et al. systematically investigated the influence of basic physical parameters of molecules on the energy charge transfer process of target molecules in drugs (Chen et al., 2005b; Chen et al., 2007d; Li et al., 2011; Li et al., 2011; Li et al., 2012; Li et al., 2013; Badu-Tawiah et al., 2013; Li et al., 2013; Chen et al., 2014; Xu et al., 2014). As shown in Table 1, the results were as follows:

1. The polar of the drug molecule is closely associated with the interaction between the drug molecule and the energy carrier. When the drug molecule interacts with energy/charge carrier, ions thus are produced. It has been known that the polar compounds have an uneven charge distribution and a large electron pair offset. Therefore, it is easy to give the electron pair to one side, resulting in ionization of the compounds. For non-polar molecules, EESI-MS can use charged cations or anions in the solution to adsorb on the polar group of non-polar molecules to generate molecular ions. Therefore, EESI-MS can be used for the detection of polar/non-polar substances.
2. According to Brønsted-Lowry proton theory, acid dissociation constant (Ka) of an acid HA is expressed by the equation: Ka = [A^−^][H^+^]/[HA], whereas base dissociation constant (Kb) of a base B is expressed by the equation: Kb = [HB^+^][OH^−^]/[B]. Acidic drug with the larger Ka and the smaller pKa typically shows a stronger acidic intensity and a higher ionization ability. When the interaction of molecules is biased towards the deprotonation state, the Ka value increases, and the pKa value decreases, so the stronger the acid strength, the easier it is to be ionized. On the contrary, when the molecular action is in the protonated state, the Ka value decreases, the pKa value increases, and the ionization strength becomes weaker. Similarly, alkaline drug with the larger Kb and the smaller pKb generally shows a stronger alkaline intensity and a higher ionization ability. At the same time, the influence of polarity, proton affinity potential, ionization energy and other factors of the drug should be considered comprehensively.
3. When there is a strong interaction between the energy charge carrier and the target molecule, the more energy transferred, the more it can promote the ionization of the target molecule. Therefore, the sensitivity of the instrument detection is increased.
4. When there is an extraction process in the energy-charge transfer, the law of similar phase dissolution still applies, so that the selectivity of ionization can be improved.

**TABLE 1:** Application of EESI-MS in pharmaceutical chemicals analytical evaluation.

Polarity	Drug type	Representative	Ionization sources	Molecular structural formula	Intensity of acid and alkali	Ion form	LOD (µg/L)	Reference
Strong polarity	Sedative hypnotics	Phenobarbital	EESI-MS	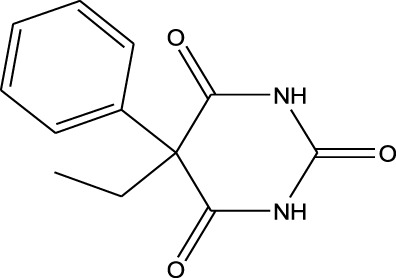	pKb6.6	[M + H]^+^	0.049	Liu et al. (2019)
	Analgesic drugs	Morphine(MOP)	EESI-MS	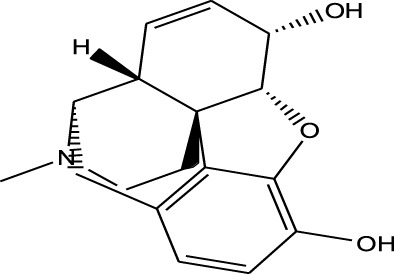	pKb5.8	[M + H]^+^	0.031	Liu et al. (2019)
	Anti-angina drugs	Nitroglycerin	ND-EESI-MS	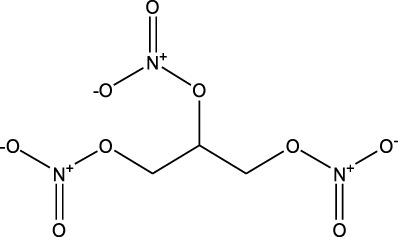	—	[M-AC]^-^	—	Chen et al. (2009)
	Tetracyclines	Tetracycline	ND-EESI-MS	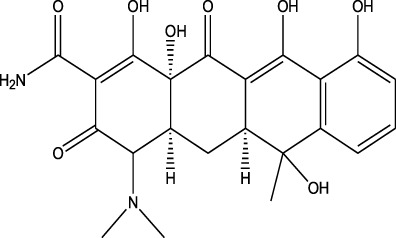	pKa3.8	[M + H]^+^	1. 080	Deng et al. (2016)
	Chloramphenicols	Chloramphenicol	ND-EESI- MS	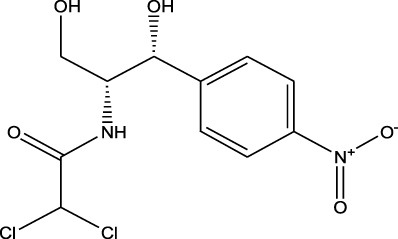	pKb4.0	[M-H]^-^	—	Huang et al. (2014)
	Cough medicine	Codeine	EESI-MS	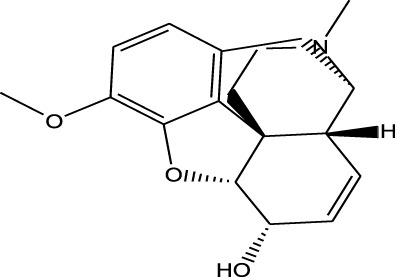	pKb5.8	[M + H]^+^	0.031	Liu et al. (2019)
	Benzodiazepines	Nitrazepam	EESI-MS	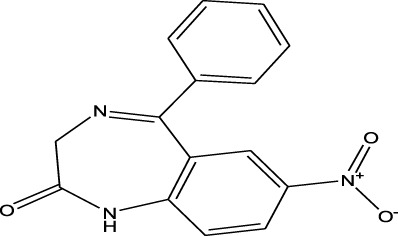	pKb2.3	[M + H]^+^	—	Liu et al. (2019)
	Methylxanthines	Caffeine	EESI-MS	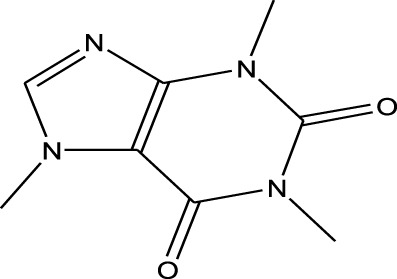	pKb3.6	[M + H]^+^	0.140	Liu et al. (2019)
	Opiate agonist drugs	Demerol	EESI-MS	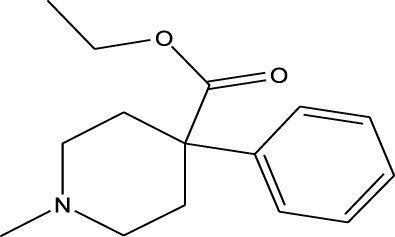	pKb6.8	[M + H]^+^	0.034	Liu et al. (2019)
	Antihistamines	Promethazine	EESI-MS	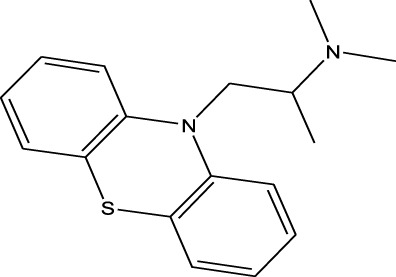	pKb4.9	[M + H]^+^	0.018	Liu et al. (2019)
	Alpha-and Beta-adrenergic Agonists	Ephedrine	EESI-MS	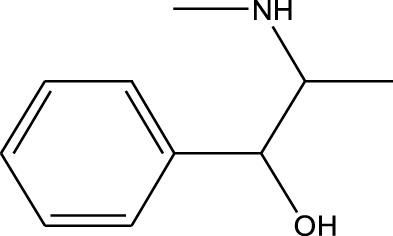	pKb3.7	[M + H]^+^	0.090	Liu et al. (2019)
Polarity	Benzodiazepines	Triazolam	EESI-MS	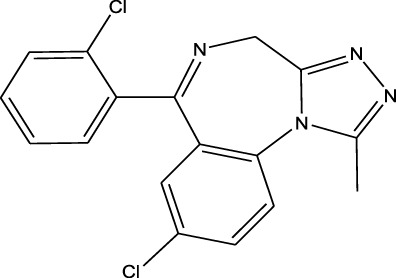	pKa4.3	[M + H]^+^	0.023	Liu et al. (2019)
	Fluoroquinolones	Norfloxacin	EESI-MS	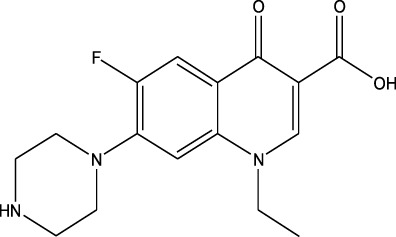	pKb5.2	[M + H]^+^	—	Li (2015)
	Sulfonamides	Sulfamethazine	ND-EESI-MS	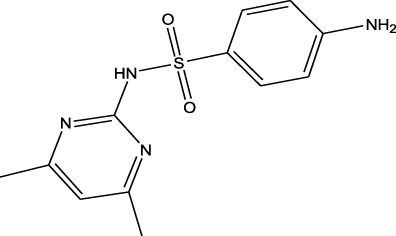	pKb6.4	[M + H]^+^/[M + Na]^+^	0.010	Liu et al. (2011b)
	Sulfonamides	Sulfafurazole	ND-EESI-MS	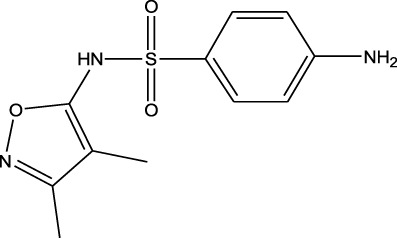	pKa2.2	[M + H]^+^	—	Liu et al. (2011b)
	Sulfonamides	Sulfathiazole	ND-EESI-MS	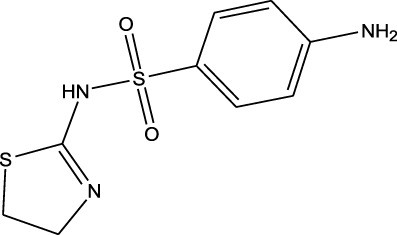	pKb6.8	[M + H]^+^	0.010	Liu et al. (2011b)
	Sulfonamides	Sulfadiazine	ND-EESI-MS	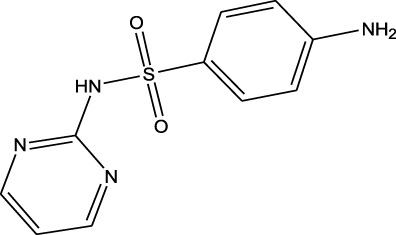	pKa6.4	[M + H]^+^/[M + Na]^+^	—	Liu et al. (2011b)
	Sulfonamides	Sulfapyridine	ND-EESI-MS	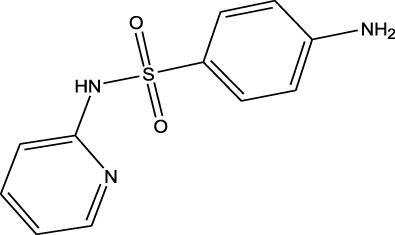	pKb6.6	[M + H]^+^/[M + Na]^+^	0.10	Liu et al. (2011b)
	Analgesic drugs	Paracetamol	ND-EESI-MS	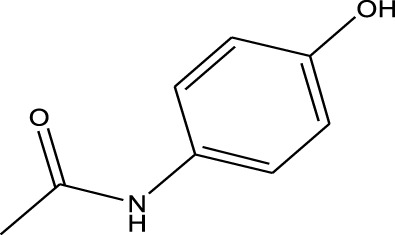	pKb4.2	[M + H]^+^	—	Williams and Scrivens, (2008)
	β-receptor antagonists	Propranolol	EESI-MS	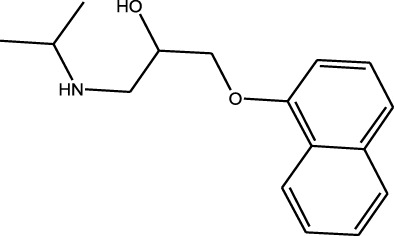	pKb4.3	[M + H]^+^	—	Koyanagi et al. (2015)
Weak polarity	Benzodiazepines	Diazepam	EESI-MS	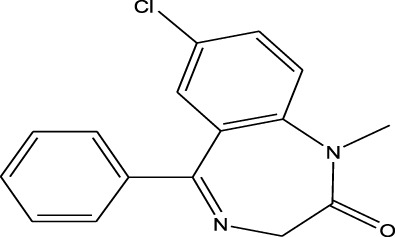	pKa3.4	[M + H]^+^	0.0024	Liu et al. (2019)
	Sulfonamides	Sulfanilamide	ND-EESI-MS	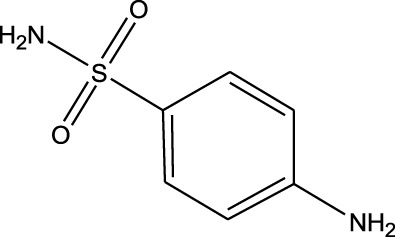	pKb3.4	[M + H]^+^/[M + Na]^+^	0.10	Liu et al. (2011b)

**FIGURE 3:**
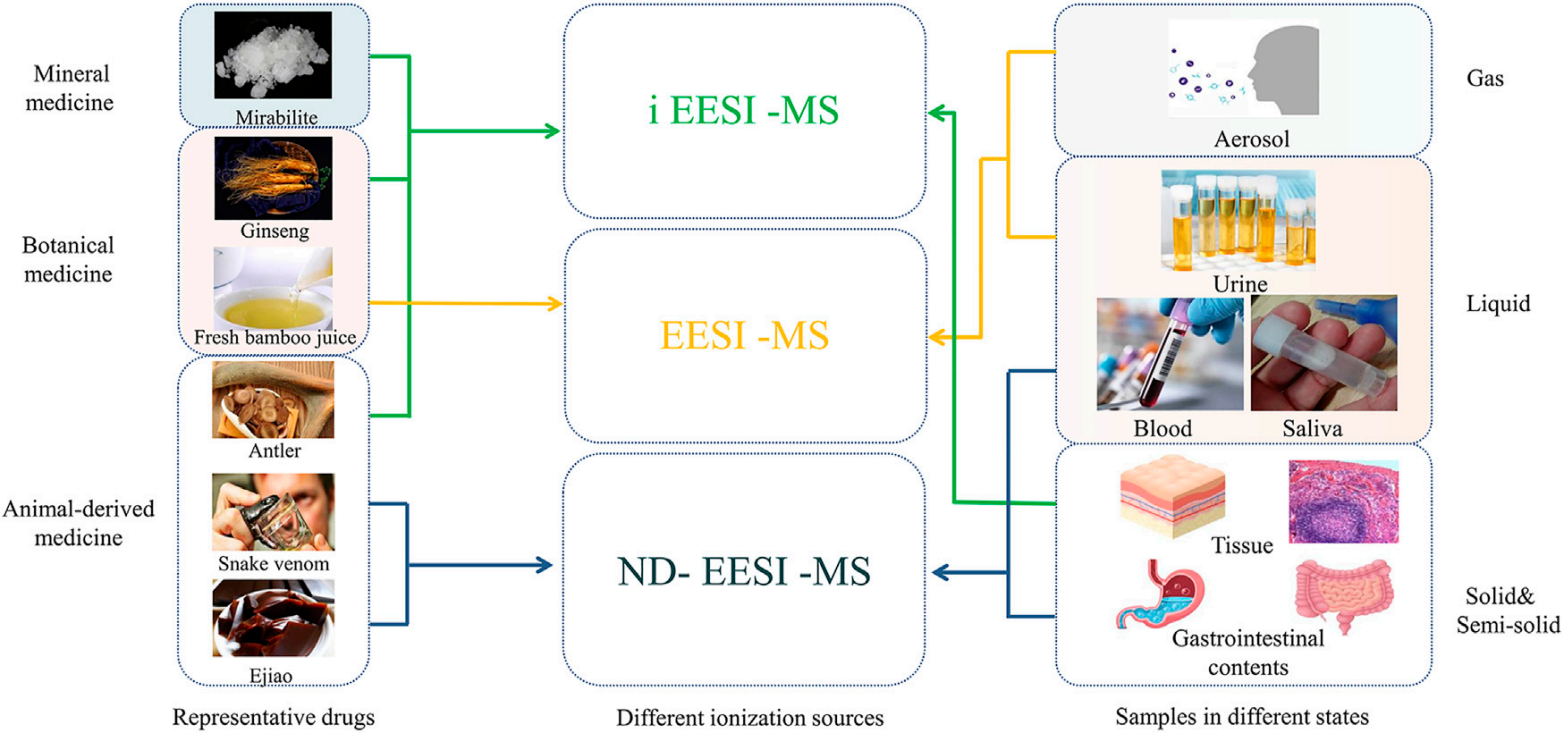
Selection of ionization sources based on traditional Chinese medicine and different sample states.

Due to the different dosage states, drugs usually exist in the state of solid, liquid, colloidal, gaseous and heterogeneous mixtures. Therefore, suitable ionization variants of EESI can be selected according to the different states. As shown in Table 2, for Liquid preparation (injection, eye drop, tincture, etc.), EESI can directly ionize the sample without pretreatment, which is ideal for real-time online analysis (Chen et al., 2007b; Chen and Zenobi, 2008; Ding et al., 2009; Law et al., 2009; Chen et al., 2010; Zhang et al., 2010; Li et al., 2011; Zhang et al., 2013; Xu and Chen, 2018). Rapid, sensitive and reliable analysis of metabolic components in biological samples is vitally important, which is commonly used in pharmacokinetic studies. In the study of drug metabolism with urine samples for example, the ionization sources selection is also shown as the same as above (Figure 3). For example, urine and other liquid samples (Huang et al., 2007) can be directly introduced to EESI for ionizatin and post mass analysis. Zhong et al. analyzed phospholipids in the urine of HCC, cirrhosis, chronic hepatitis B and healthy controls by EESI, which showed the strongest signal peak at voltage +3 kV, methanol/double vapor/glacial acetic acid (1/1/0.02), and the highest signal peak in the range of *m/z* 50 to 1,000 (Zhong, 2020). EESI also has the outstanding ability to directly analyze volatile and nonvolatile compounds in gaseous samples. Wang et al. adopted the EESI-MS/MS method to conduct a semi-quantitative analysis of the creatinine content in the exhaled gas of patients with kidney disease, and found that the more serious the kidney damage was, the higher the creatinine content in the exhaled gas was (Wang et al., 2015).

**TABLE 2:** Selection of ionization sources for the analysis of drugs in different dosage forms.

Drug classification	Western medicine				Traditional Chinese medicine			
Morphology	Solid preparation	Semi-solid preparation	Liquid preparation	Gas preparation	Solid preparation	Semi-solid preparation	Liquid preparation	Gas preparation
Dosage form category	Tablet, suppositories, pill	Ointment, syrup	Aromatic water, injection, eye drop, lotion, tincture, soft capsule	Aerosol, powder mist	Tablet, pill, suppositories, plaster	Syrup, fluid paste, ointment	Decoction, wine, dew, injection	Aerosol, powder mist
Ionization Sources	iEESI	ND-EESI	EESI	EESI	iEESI	ND-EESI	EESI	EESI

EESI has been also applied for traditional Chinese medicine samples (as shown in Figure 3). Firstly, EESI-MS can be used for screening of endogenous substance. For example, (Lai et al., 2022) conducted a self-constructed real-time EESI-MS device to investigate the compatibility of aconite and meat cooking and attenuating toxicity, and analyzed 9 diester, monoester and alcohol-amine aconitine compounds and 9 internal standard compounds, and they found no mass spectrum response saturation and good resistance to complex matrix interference, both of which could meet the requirements of quantitative analysis of components. Furthermore, EESI-MS can also be used for the identification of exogenous substances in traditional Chinese medicine. During the cultivation of Chinese medicinal materials, pesticides, heavy metals, sulfur dioxide and other exogenous harmful substances are introduced due to improper fertilizer application or environmental pollution and improper operation in the processing and storage. It has been reported that multistage extraction electrospray ionization mass spectrometry (EESI-MS^n^) rapidly detected trace lead levels in an aqueous solution, with a detection limit (LOD) of 10^−13^ g/mL, indicating that EESI-MS is a useful tool for the rapid detection of lead in various solutions, which shows the promise of the rapid screening of lead-contaminated aqueous liquid samples (Liu et al., 2012).

ND-EESI can desorbate a small amount of samples with suitable neutral airflow, and separate the sampling and ionization process from time and space, which has been widely applied to the analysis of sticky samples such as paste, colloid and syrup. This method is particularly suitable for mass spectrometry in harsh environment such as high temperature, low temperature, biohazard or radioactivity (Figure 3). (Chen et al., 2007b; Chen and Zenobi, 2008; Ding et al., 2009; Law et al., 2009; Li et al., 2011; Liu et al., 2011; Deng et al., 2017). Huang et al. (2014) used ND-EESI to detect chloramphenicol (CAP) in honey. By using heated methanol-N_2_ as spray reagent, they observed that the CAP limit of determination (LOD) reduced from 73.3 to 0.3 ng/mL, and the CAP detection was linear in the range of 1–5,000 ng/mL (R = 0.9947). This study showed that ND-EESI-MS is powerful for direct, rapid, and quantitative CAP analysis in honey samples with high sensitivity, precision, and specificity . In addition, ND-EESI can be selected for desorption of blood, sputum, intestinal contents and other samples due to their viscosity. ND-EESI-MS and collision induced dissociation (CID) were reported to detect sputum metabolites from 143 spontaneous sputum samples. In this study, 19 altered metabolites were detected by ND-EESI-MS from 76 cases of LAC and 67 cases of control (Zheng et al., 2021). Moreover, ND-EESI technology can also be used for the detection of exogenous substances. For example, a previous study using ND-EESI without any sample pretreatment reported that there were three kinds of organophosphorus insecticides and two kinds of carbamate insecticides present in honey. The detection limits of the assay ranged from 1.16 to 4.18 ng/g for the studied pesticides. The developed assay was linear over the concentration range of 20.00-1000.00 ng/mL, with correlation coefficients of more than 0.996, and the recoveries ranged between 87.00% and 114.98%, with RSD lower than 6.22% for all pesticides (Deng et al., 2017).

iEESI can achieve good sensitivity for solid samples (such as tablet, pill, etc.) (Zhang et al., 2013; Xu and Chen, 2018), as well as the samples of heart, liver, spleen, lung and kidney (Figure 3) (Lu et al., 2020; Wu et al., 2021). Lu used iEESI-MS to conduct a comparative study on the morphological changes of phospholipids in human and mouse liver cancer, and they found that iEESI-MS is a promising technology for the rapid targeted diagnosis of liver cancer with high accuracy, sensitivity and specificity (Lu et al., 2020).

## 4 Application of EESI-MS in pharmaceutical chemicals synthetic preparation

EESI can monitor chemical reaction online. Briefly, the reactor was a three necked round bottom flask placed on a magnetic stirrer. A 50 mL flask was used as a safety bottle to prevent the solution from backflow. The gas flowmeter and glass rotor were used to regulate the flow of carrier gas (N_2_). Generally, N_2_ was introduced into the reaction vessel through glass tube. The reaction products and intermediates were carried and sprayed out of a capillary by N_2_. Another sprayer generates charged spray solvent droplets. The analytes were extracted by solvent droplets for further mass spectrometry analysis (Marquez et al., 2008; Zhu et al., 2008; Zhang et al., 2019).

**FIGURE 4:**
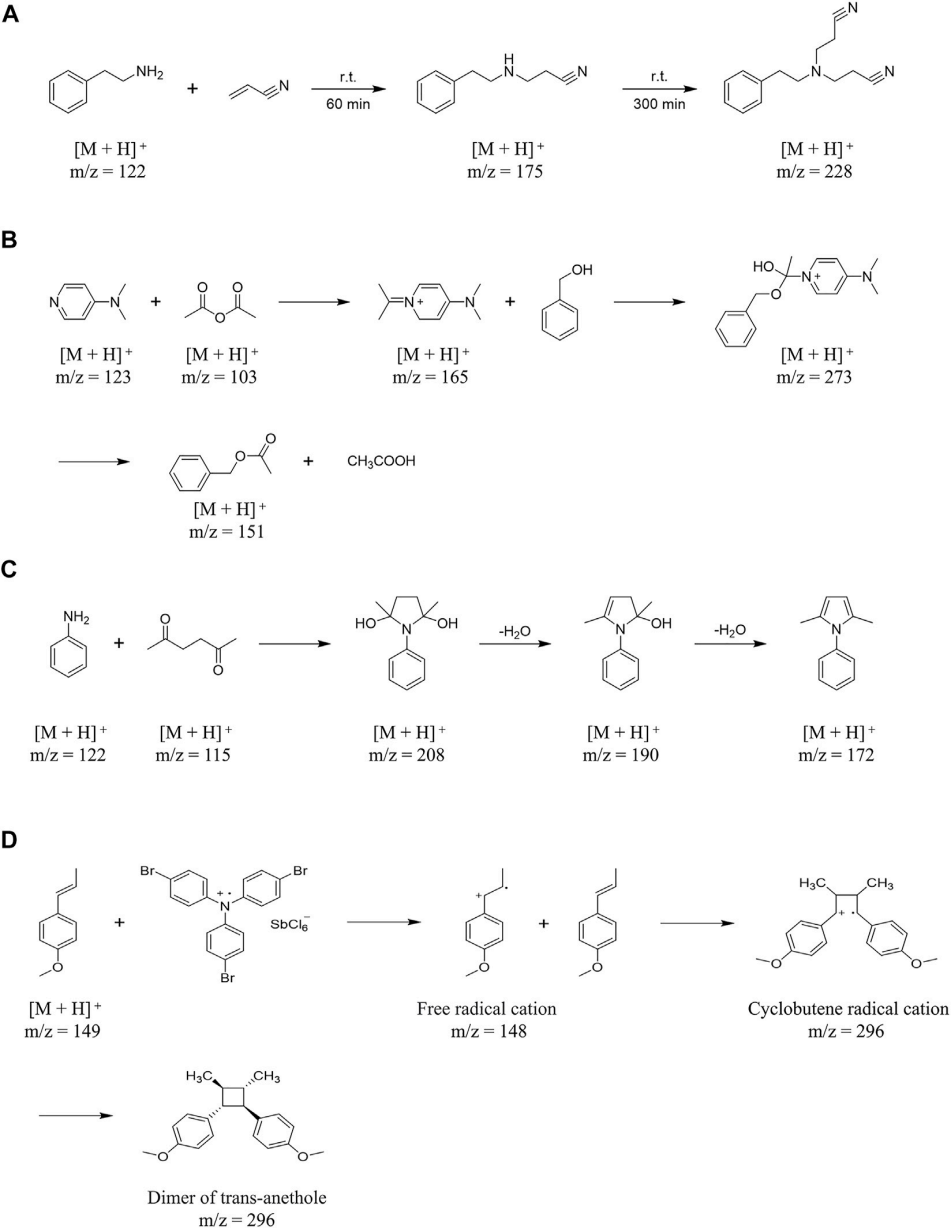
The mechanistic analyses of several different types of reactions by EESI. **(A)** The mechanism of Michael addition reaction of phenylethylamine (PEA) and acrylonitrile in ethanol. **(B)** The reaction mechanism of catalytic acetylation of acetic anhydride and benzyl alcohol in the presence of 4-DMAP. **(C)** The mechanism of acetic acid-catalyzed reaction between aniline and acetonylacetone. **(D)** The mechanism of dimerization of trans-anethole based on free radical cation cyclization reaction.

EESI has been reported to be used for the mechanistic analyses of several different types of reactions. For example, Zhu et al. (2008) used EESI to real-time monitor the one-step Michael addition reaction of phenylethylamine (PEA) and acrylonitrile in ethanol. In this reaction, the delay between the changes in solution and the corresponding signal was estimated to be in the range from 0.2 to 1 s. Phenylethylamine (10.4 mL) and acrylonitrile (12.5 mL) are easy to Michael addition reaction when stirred in ethanol (27 mL), which can react at room temperature. During this reaction, phenylethylamine propiononitrile (PEAP, *m/z* 175) can obtained in a short time, but after a longer reaction time, 3-[(2-cyanoethyl)phenylethylamino]-propionitrile (CPEAP, *m/z* 228) can be produced by adding acrylonitrile to PEAP. EESI effectively monitors PEAP and CPEAP at 60 and 300 min, respectively (Figure 4A) (Zhu et al., 2008).

In addition, Zhu et al. (2008) also used EESI to real-time monitor the multi-step acetylation reaction of benzyl alcohol and acetic anhydride catalyzed by 4-dimethylaminopyridine (4-DMAP) in dichloromethane. In this study, they proposed a possible reaction mechanism about the catalytic acetylation of acetic anhydride and benzyl alcohol in the presence of 4-DMAP, and found the key intermediate protonated benzyl acetate (*m/z* 165) which support for this assumption (Figure 4B) (Zhu et al., 2008). Similarly, Zhang et al. 2019 used EESI to real-time monitor the reaction process of aniline and acetylene acetone catalyzed by acetic acid, and proved that acetylene acetone can generated three different forms of ions in the reaction: Acetonylacetone molecular ion at *m/z* 115, radical of acetonylacetone combined with water [M + H_2_O]^+^· at *m/z* 132 and acetonylacetone sodiated [M + Na]^+^ ion at *m/z* 137, and then condensed and cyclized with aniline, with the removal of two molecules of water to obtain the product (Figure 4C) (Zhang et al., 2019).

Furthermore, EESI can be used as a tool for the detection and characterization of reactive intermediates of reactions in solution. Marquez et al. (2008) observed and characterized the dimerization of trans-anethole based on free radical cation cyclization reaction by using EESI, and detected directly from the ongoing reaction in solution. This demonstrated the ability of EESI-MS to study rapid reactions in solutions within a millisecond time window (Figure 4D).

## 5 Conclusion

EESI-MS is a technique elaborately developed for liquid, solid, colloidal, powder, paste and other samples that takes advantage of its high sensitivity, selectivity, accuracy and rapidity. With different samples and sampling methods, EESI-MS have been developed for advanced bioanalytical and life science applications, including but not limited to the analysis of various pharmaceutical compounds, different pharmaceutical preparations, and herb medicine materials, and drug synthesis.

In the future, EESI-MS will be further developed in several aspects, including: 1) *In vivo* analysis: The EESI device will be further optimized to improve quantitative performance in living objects analysis, which makes EESI the preferable technique over others for *in vivo* analysis, including but not limited to biologically living objects, *in vivo* drug analysis, pharmaceutical dynamics, *in vivo* metabolomics, etc.; 2) Reliability: The analysis process will be further strengthened, and the standardized analysis scheme will further shortened the analysis time; 3) Field application: Combined with miniature mass spectrometer, dedicated EESI-MS instruments will be developed for rapid field analysis of public safety (food safety, environmental testing, criminal investigation safety, etc.) and life safety (early diagnosis of disease, precision treatment of disease, and postoperative health monitoring). On the basis of a deep understanding of the basic process of ion formation, it is of great significance to establish the EESI-MS analysis technology and method system, improve the mass spectrometry analysis effect of complex matrix samples, and expand the existing means of drug analysis and preparation, which will promote the development of mass spectrometry and related disciplines in various fields.
